# Distortion of Near-Surface Seawater Temperature Structure by a Moored-Buoy Hull and Its Effect on Skin Temperature and Heat Flux Estimates

**DOI:** 10.3390/s90806119

**Published:** 2009-07-31

**Authors:** Yoshimi Kawai, Kentaro Ando, Hiroshi Kawamura

**Affiliations:** 1 Japan Agency for Marine-Earth Science and Technology, 2-15 Natsushima-Cho, Yokosuka, 237-0061, Japan; E-Mail: andouk@jamstec.go.jp (K.A.); 2 Center for Atmospheric and Oceanic Studies, Graduate School of Science, Tohoku University, Aoba-ku, Sendai 980-8578, Japan; E-Mail: kamu@ocean.caos.tohoku.ac.jp (H.K.)

**Keywords:** moored buoy, sea surface temperature, diurnal warming, heat flux

## Abstract

Previous studies have suggested that the accuracy of temperature measurements by surface-moored buoys may be affected by distortions of the near-surface temperature structure by the buoy hull on calm, sunny days. We obtained the first definite observational evidence that the temperature near the hull was not horizontally homogeneous at the same nominal depth. We observed large temperature differences of 1.0 K or more between thermometers at 0.2 m depth. The distortion of the surface temperature field yielded an error in estimates of daytime net surface heat flux up to more than 30 Wm^−2^.

## 1. Introduction

Diurnal variation is one of the dominant components in the variation of sea surface temperature (SST). Although the diurnal SST variation has often been neglected in oceanography and meteorology, recently many researchers are giving it greater attention (for a review see [1]). For example, Clayson and Chen [2] investigated the effect of the diurnal SST variation on the atmosphere during the Tropical Ocean Global Atmosphere/Coupled Ocean-Atmosphere Response Experiment (TOGA/COARE) using a coupled single-column model. They showed that the diurnal variation could change the vertical profiles of air temperature, moisture content, and abundance of clouds. Johnson *et al.* [3] analyzed atmospheric sounding data from TOGA/COARE and found that large ranges in diurnal SST cycles caused diurnal cycles in the atmospheric mixed layer on calm days. The inclusion of the upper-ocean diurnal cycle in a coupled ocean-atmosphere general circulation model affected the predictive ability of the model and the predicted strength of the Madden-Julian Oscillation [4,5]. Yasunaga *et al.* [6] showed that observed afternoon increases in precipitable water and radar echo coverage corresponded to large diurnal rises of skin SST (SST_skin_) in the tropical Indian Ocean. However, they also indicated that the increase in latent heat flux could not account for the increase in precipitable water. The processes by which the diurnal SST variation affects the atmosphere have not yet been clarified, and observational studies along with model studies are still indispensable. However, there can be problems with one of the most common measurements in oceanography–the *in situ* SST–when the diurnal surface warming is strong.

Currently five kinds of SST are strictly defined (see Table 1 in [1]) to encourage reporting of the measurement depth along with the temperature because the temperature can change drastically with depth when a diurnal thermocline forms. Most *in situ* observations reported as “SST” are in fact temperatures at depth (SST_depth_), which were observed at depths of 1 m or greater. Large diurnal SST increases are always accompanied by a shallow diurnal thermocline, which can be shallower than 1 m under strong warming conditions. Hence, it is necessary to know the detailed vertical temperature structure near the surface, and not only SST_depth_, to investigate the air-sea interactions associated with diurnal SST variation. However, previous research [7,8] suggested that the temperature field around surface buoys, such as those used for measuring *in situ* SST, might be distorted by the buoy hull, because buoy-observed temperatures at depths of 1 m or 1.5 m were too high compared with the values estimated from the surface heat budget when a diurnal thermocline formed. Since the authors did not determine the two-dimensional temperature structure around a buoy hull, they could not confirm this hypothesis completely.

In this study we attached additional recording thermometers to specific locations on the hull of a moored buoy to obtain detailed measurements of seawater temperatures around the hull and to determine whether the temperature field around the hull was distorted. This study is necessary to allow accurate measurements of diurnal SST variations, and to improve the estimation of the air-sea heat flux.

## 2. Method

**Figure 1.**
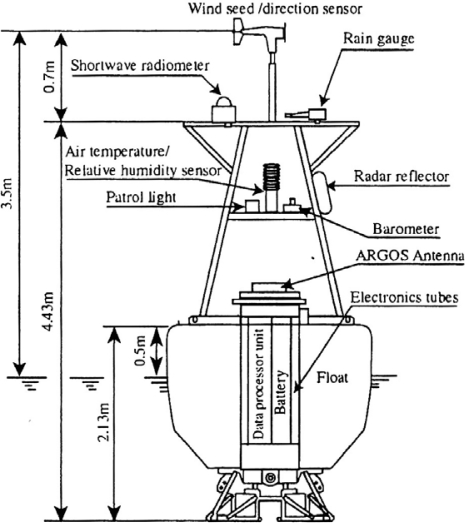
Schematic of the TRITON buoy.

The Japan Agency for Marine-Earth Science and Technology (JAMSTEC) deploys and maintains the moored buoys of the Triangle Trans-Ocean buoy Network (TRITON) in the western tropical Pacific and the eastern Indian Oceans. The hull of the TRITON buoy has a diameter of 2.4 m (Figure 1). A conductivity/temperature (CT) sensor for standard SST measurement is installed on the bottom of the buoy hull at 1.5 m depth. This is referred to as SST_1.5m_. The buoy has a 2.3 m high tower equipped with meteorological sensors for wind speed and direction, air temperature, relative humidity, atmospheric pressure, shortwave radiation, and precipitation [8]. Since the TRITON buoys do not observe downward longwave radiation, we used an empirical bulk formula (see Appendix in [9]) to estimate longwave radiation for use in calculating SST_skin_ in Section 3.3.

**Figure 2.**
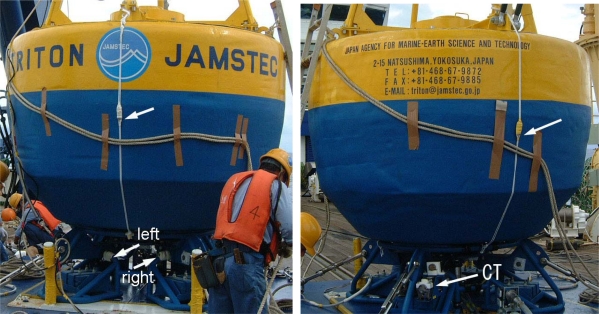
Photograph showing the hull of the TRITON buoy and the specially attached thermometers on the “mark” side (left photograph) and the “opposite” side (right photograph). The boundary between yellow and blue sections of the hull indicates the water-line. The white arrows point to the thermometers. The upper two thermometers were attached using white ropes.

In addition to the regular CT sensor, we installed four small thermometers on the hull of a buoy that was deployed at 0°N, 156°E for about one year beginning in February 2005 (one thermometer stopped working on 1 June 2005). Each extra thermometer contained a thermistor, a data logger, and a battery. The thermometer types and their locations on the buoy are summarized in Table 1 and shown in Figure 2. One of the four was of a different type and its measurement interval was 15 min., whereas the others were able to record temperatures every 2 minutes because of their higher logger capacities. Two thermometers were attached to the side of the hull at 0.2 m depth, and the other two were on the bottom of the hull at 1.5 m depth. One of the upper two thermometers on the side of the hull with the JAMSTEC logo (“mark” side), and the other was on the exact opposite side (“opposite” side) (Figure 2). To protect the thermometers from shocks and direct insolation, they were enclosed in small stainless-steel containers with holes. We calibrated all of the thermometers against a standard, and confirmed that they were all accurate to within ±0.05 K.

## 3. Results and Discussion

### 3.1. Temperature Variations on a Short Time Scale

**Figure 3.**
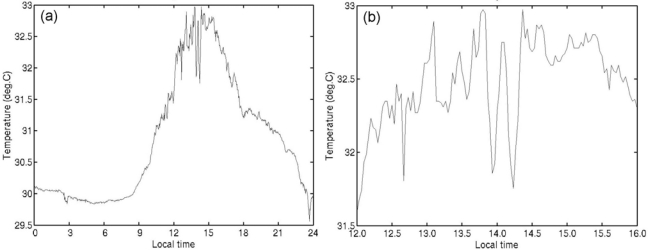
(a) Example of the temperature variation at 0.2 m depth on the mark side of the hull on 16 May 2005. (b) Enlargement of (a) showing the temperature from 1200 to 1600 LT.

We obtained long-term successive temperature data for two nominal depths with a fine temporal resolution. We first checked temperature variations on a time scale of less than one hour. For example, on 16 May 2005 the amplitude of the diurnal temperature variation at 0.2 m depth on the mark side exceeded 2 K (Figure 3). Whereas the short-term variation was small in the morning, the short-term temperature fluctuations became larger after 1200 local time (LT). This trend of larger short-term daytime temperature fluctuations associated with greater diurnal warming was seen throughout the data set.

**Figure 4.**
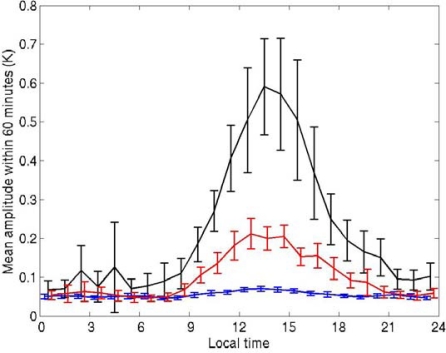
Average hourly temperature ranges at 0.2 m depth on the mark side, from 3 February 2005 to 20 February 2006. Any linear trend within each hour was removed. Vertical lines indicate 95% confidence intervals. Blue line: days with diurnal temperature amplitude <1.0 K; red line: amplitude ≥1.0 K and <2.0 K; black: amplitude ≥2.0 K.

We determined the hourly range of temperature fluctuations at 0.2 m depth as a function of local time for different ranges of diurnal amplitude (Figure 4). The diurnal amplitude was defined as the difference between the maximum temperature recorded between 0900 and 2100 LT and the minimum before 0900 LT, and was calculated using hourly mean values. The linear trend within each hour was removed when we calculated the hourly fluctuations, which were defined as the differences between the maximum and the minimum within each hour.

The fluctuations before 0800 LT were less than 0.1 K and were not dependent on the diurnal amplitude. However, the short-term fluctuations clearly became larger during the daytime when the diurnal amplitude was large (Figures 3 and 4). This pattern was also seen for the temperature at 1.5 m although the fluctuations were slightly smaller (not shown). This means that the temperature near the surface could change substantially over a short period when a diurnal thermocline forms. Our observations indicate that the shallow, stable thermal stratification repeatedly decayed and re-developed over short periods in the daytime, possibly because of buoy-induced turbulence or short-term fluctuations of the atmosphere. We cannot identify the reason at present.

### 3.2. Position-Dependent Temperature Differences around the Buoy

**Figure 5.**
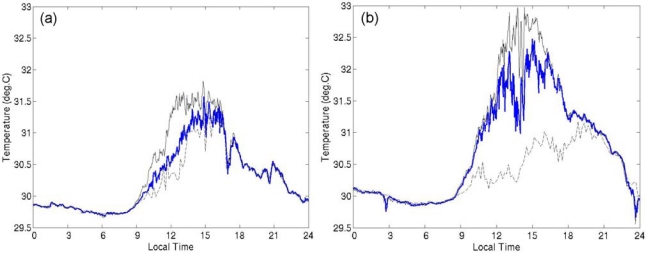
Temperature variations at 0.2 m depth on (a) 8 April 2005 and (b) 16 May 2005. Solid black line: measurements on the mark side of the hull; blue line: opposite side; broken black line: CT sensor at 1.5 m depth. The sampling interval of the CT sensor was 10 minutes; the others were 2 minutes.

**Figure 6.**
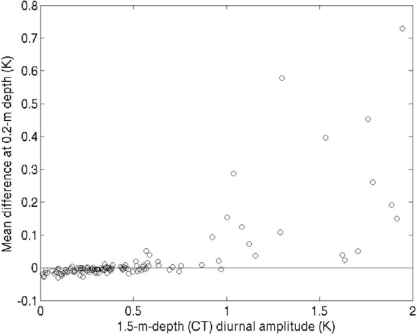
Temperature differences between the mark side and the opposite side at 0.2 m depth as a function of the diurnal amplitude of the CT temperature at 1.5 m from 3 February 2005 to 31 May 2006. The temperature differences were averaged between 1100 and 1500 LT.

We found that the daytime seawater temperature varied substantially with position around the buoy hull when the diurnal warming was large (Figure 5). In the daytime the temperatures at 0.2 m depth were higher than those at 1.5 m depth. This difference was presumably due to a diurnal thermocline formed between 0.2 m and 1.5 m. Furthermore, there were also temperature differences exceeding 1.0 K between thermometers on opposite sides of the buoy at the same 0.2 m depth on several days. We compared the temperature differences between the mark side and the opposite side at 0.2 m to the diurnal amplitude observed with the CT sensor at 1.5 m depth (Figure 6). When the diurnal amplitude was less than 1.0 K, most of the temperature differences at 0.2 m were less than ±0.05 K. The temperature difference at 0.2 m became substantial when the diurnal warming became greater. The mark-side temperature tended to be higher than the opposite-side temperature. Although we do not know in which direction the buoy hull faced during these observations, the results suggest that the mark-side thermometer was mainly on the upstream side and/or the sunny side.

**Figure 7.**
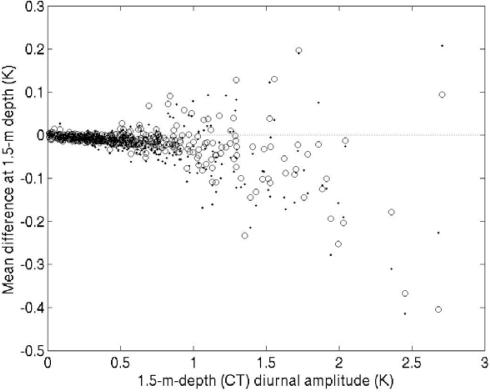
Temperature differences at 1.5 m between the right-side thermometer and the CT sensor (dot) and between the left-side thermometer and the CT sensor (circle) as a function of the diurnal amplitude of the CT temperature. The temperature differences were averaged between 1100 and 1500 LT. For the right-side thermometer, temperatures at 10-min intervals were calculated by linear interpolation. The temperatures measured by the left-side thermometer were re-sampled at 10-min intervals.

There were also differences among the daytime temperatures measured at 1.5 m depth by the specially attached thermometers and the CT sensor (Figure 7). The temperatures measured by both the right and left thermometers deviated from that observed with the CT sensor, and the deviations increased with stronger diurnal warming. Both of the thermometers attached at 1.5 m depth on the mark side (Figure 2) tended to record lower temperatures than the CT sensor. On the other hand, the temperature recorded at 0.2 m on the mark side was higher than that on the opposite side (Figures 5 and 6). When a thermocline is formed above 1.5 m depth, the temperature rise at 1.5 m is suppressed because the downward turbulent heat transport is prohibited [7,8]. Hence, the temperature at 1.5 m will be lower on the side where the diurnal thermocline is sharper. The vertical temperature gradient on the opposite side would be weaker, possibly because of eddies induced by the buoy hull. As another possibility, the warmer water heated by insolation might have stagnated on the sunny side in the lateral boundary layer around the hull, and the 0.2-m mark-side thermometer measured this higher temperature.

Unfortunately, we could not identify the positions of the sensors relative to the current direction because the TRITON buoys are not designed to record the azimuth angle of the buoy hull. We would need to improve the buoy instrument system to record the azimuth angle in order to investigate the detailed three-dimensional temperature structure around the buoy hull from the viewpoint of a fluid dynamics problem of stratified flow past a body. However, we do not need it now because our purpose in this study is to obtain observational evidence that there is temperature difference around the buoy hull even at the same depth.

### 3.3. Effects on Surface Heat-Flux Estimates

**Figure 8.**
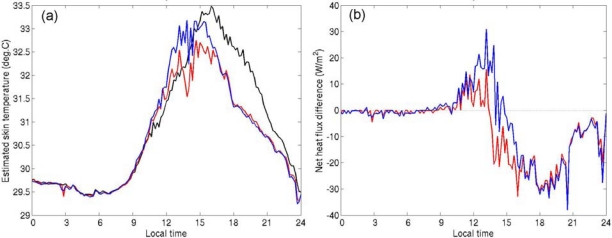
(a) Skin temperatures (SST_skin_) estimated from the 0.2-m-depth mark-side temperature (blue), from the 0.2-m-depth opposite-side temperature (red), and from the 1.5-m-depth CT temperature (black) using the warm-layer and cool-skin models [10] on 16 May 2006. Temperature data from 0.2 m were re-sampled every 10 minutes. (b) Differences between the net heat flux estimated from the 0.2-m-depth mark-side temperature and that from the CT temperature (blue), and between the net heat flux estimated from the 0.2-m-depth opposite-side temperature and that from the CT temperature (red) on 16 May 2006.

We evaluated the effect of the temperature differences around the hull on surface heat-flux estimates. We calculated SST_skin_ and surface heat flux from the 0.2-m-depth temperatures and the CT-measured 1.5-m-depth temperature by using the cool-skin and warm-layer models and the bulk parameterization proposed by Fairall *et al.* [10,11]. Figure 8 shows an example of the estimated SST_skin_ and the differences in surface heat fluxes estimated from the three temperatures recorded on 16 May 2006 (Figure 5b). The differences among the three estimates of SST_skin_ exceeded 1.0 K in the daytime. The SST_skin_ estimated from the 1.5-m-depth CT temperature was highest in the afternoon. This might be due to the platform effect described by [7,8], who suggested that the buoy CT sensor measures a temperature higher than the true temperature at its nominal depth when a diurnal thermocline has formed.

**Figure 9.**
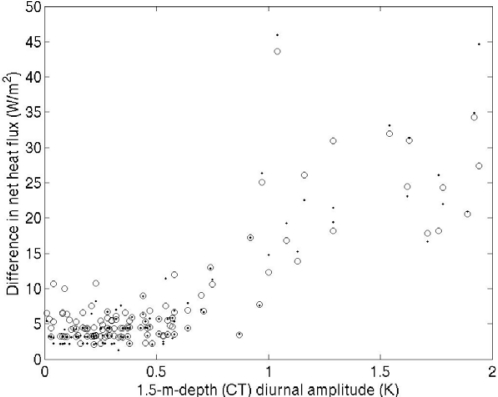
Differences between the net heat flux estimated from the 0.2-m-depth mark-side temperature and that estimated from the CT temperature (circle), and between the net heat flux estimated from the 0.2-m-depth opposite-side temperature and that estimated from the CT temperature (dot) from 3 February 2005 to 31 May 2006 as a function of the diurnal amplitude of the CT temperature. The differences were averaged between 1100 and 1500 LT.

The surface heat fluxes shown in Figure 8b were the sums of latent heat flux, sensible heat flux, and the upward longwave radiation calculated from the SST_skin_ and the meteorological data obtained at the buoy. In this case, the daytime heat fluxes estimated from the 0.2-m-depth temperatures deviated from that estimated from the CT temperatures by more than 10 Wm^−2^, and the deviation sometimes reached 30 Wm^−2^. The difference between the mark-side and opposite-side heat-flux estimates also at times exceeded 10 Wm^−2^. We compared the differences in heat-flux estimates over the course of this study as a function of the diurnal amplitude of the CT temperature (Figure 9). When the diurnal amplitude at 1.5 m exceeded 1.0 K, the difference between daytime heat-flux estimates based on temperatures at 0.2 m and 1.5 m often exceeded 20 Wm^−2^.

Ward [12] and Fairall *et al.* [10] indicated that the temperature difference across the warm layer can induce a difference of 50–60 Wm^−2^ in the estimated net heat flux in the daytime. Our results from this study (Figures 8 and 9) show that the effect of the hull-induced temperature distortion on the heat flux estimation can be comparable to the effect of the warm layer itself. This means that the careful SST measurement without distorting the near-surface stratification is essentially important as well we the improvement of the warm-layer modeling for the diurnal variation research.

## 4. Summary and Conclusions

With the current interest in the effects of diurnal sea surface warming on the atmosphere, there is a strong need for precise near-surface temperature measurements. Previous studies have suggested that the accuracy of temperature measurements by surface-moored buoys may be affected by distortions of the near-surface temperature structure by the buoy hull on calm, sunny days when a sharp thermocline forms at depths of less than a few meters. However, this has not been confirmed by field observations. We attached special recording thermometers to the hull of a TRITON buoy to monitor the temperature field around the buoy hull. Our observations confirmed that the temperatures field around the hull was not horizontally homogeneous at the same nominal depth, primarily when the diurnal surface warming was large. Although it was not possible to identify the reason for this inhomogeneity or to determine the detailed three-dimensional temperature structure around the hull, this result corroborates the suggestion that buoy hull distorts the temperature field.

Our observations show that the uncertainty in the *in situ* SST measurements needs not to be considered in most cases (Figure 9). If the diurnal warming is small enough, the spatial temperature differences are less than the accuracy of the thermometer, and the difference in the estimated heat flux is also negligible. However, when the diurnal amplitude of the CT temperature is greater than or equal to about 1.0 K, the difference between the net heat flux estimated from the CT temperature and that from the temperature at 0.2 m can exceed 20 Wm^−2^. Although this difference does not affect mean heat-flux estimates on a time scale of several days or more because it arises only during certain limited periods (Figure 9), this is not always negligible when quantitatively evaluating the effect of diurnal SST warming on the atmosphere [2–5].

The differences in the estimated value for SST_skin_ (Figure 8a) and the short-term fluctuations in temperature described in Section 3.1 are not negligible for the validation of satellite SSTs. These fluctuations might also be associated with the presence of the buoy hull. When the diurnal warming is strong, the temperature difference due to a time lag in a satellite-*in situ* matchup can exceed 0.5 K, which is the typical random error of satellite-derived SST, even if the time difference is less than one hour (Figure 3). A matchup of observations within a time interval of about 10 minutes or less would be desirable for the validation of satellite SST.

This study was not intended to invalidate the use of the TRITON buoy for surface observations, but rather our results should provide useful insights when developing moored buoys and other platforms that specialize in observations of heat flux and air-sea interactions associated with diurnal SST warming. The distortion of the surface temperature field is expected to depend on the size and shape of the buoy hull. Some types of moored buoy have large hulls. For example, the hull of the National Data Buoy Center (NDBC) discus buoys are 3, 10, or 12 m in diameter, larger than the TRITON buoy hulls. NDBC has also 6 m boat-shaped hulls (http://www.ndbc.noaa.gov/mooredbuoy.shtml). It will be necessary to examine the effect of each type of buoy hull on the surface temperature field for the exact *in situ* temperature measurements and heat flux estimates.

The best method to measure near-surface temperature accurately without disturbing the temperature field is to use a carefully designed profiling float that ascends slowly. Ward *et al.* [13] developed an autonomous profiler named the “Skin Depth Experimental Profiler (SkinDeEP)”, which had thin temperature sensors that protruded 47 cm from the top of the body to avoid distorting the near-surface stratification. We will be also able to utilize the Argo floats, which have been deployed over the whole oceans, to measure SST accurately by improving their temperature sensor and sampling scheme. Such kinds of instrument will be useful for research on air-sea interaction associated with the diurnal variation.

## Figures and Tables

**Table 1.** Thermometers used in this study and their positions on the TRITON buoy hull.

**Nominal depth (m)**	**Side**	**Instrument type, mfr.**	**Size (L×D, mm)**	**Measurement interval (min.)**	**Period (LT)**
0.2	Mark	MDS-Mk5/T, Alec Electronics Co., Ltd.	80 mm long × 18 mm diameter	2	2 Feb. 2005 – 21 Feb. 2006
0.2	Opposite	MDS-Mk5/T, Alec Electronics Co., Ltd.	80 mm long × 18 mm diameter	2	2 Feb. 2005 – 1 Jun. 2005
1.5	Mark (left)	MDS-Mk5/T, Alec Electronics Co., Ltd.	80 mm long × 18 mm diameter	2	2 Feb. 2005 – 21 Feb. 2006
1.5	Mark (right)	HOBO U12-015, Onset Computer Corporation	101 mm long × 17.5 mm diameter	15	2 Feb. 2005 – 21 Feb. 2006
1.5 (regular CT)	Opposite	MicroCAT, SBE37-IM, Sea Bird Electronics		10	2 Feb. 2005 – 21 Feb. 2006
